# Effects of natural deep hypothermia on cognitive abilities in mammals: hibernators and neonates

**DOI:** 10.1007/s00360-026-01662-3

**Published:** 2026-03-16

**Authors:** Richard W. Hill

**Affiliations:** Department of Integrative Biology, Michigan State University, East Lansing, MI 48824 USA

**Keywords:** Nestling rodents, Newborns, Infants, Thermoregulation, Torpor, Learning

## Abstract

Mammalian hibernators, when in hibernation, often experience deep hypothermia, defined to be a core body temperature ≤ 10 °C, from which they endogenously recover when they arouse. Similarly, among the many species of small mammals that breed in cold seasons in cold geographical areas, nestlings are vulnerable to experiencing deep hypothermia, from which they can recover by the simple expedient of being rewarmed by their parents. Both hibernation and neonatal tolerance of hypothermia are generally viewed as adaptations for mammals living in cold climates. However, following recovery from these deep-hypothermia states, there is a potential risk of cognitive dysfunction that would be maladaptive. This paper reviews the research that has been conducted on post-recovery cognitive function. Hibernation studies have focused on six rodent and two bat species – and have employed tests of spatial learning, social recognition, retention of operant conditioning, olfactory memory, and open-field habituation. Eight experiments have found that hibernators perform cognitive functions as effectively after hibernation as before, whereas five experiments have revealed degraded cognitive performance after hibernation. The specific question addressed in studies of neonates has been whether individuals exposed to deep hypothermia in the neonatal period exhibit altered cognitive function as post-weanlings or adults. Only three species have been studied, in research focused on spatial learning, taste-aversion learning, and predator evasion. All results point to no effect of neonatal deep hypothermia on later cognitive function, except for evidence that hypothermia on the first day of life (but not later days) slows spatial learning in laboratory rats.

## Introduction

Two circumstances exist in which free-living mammals experience – and can autonomously recover from – deep hypothermia, defined here to be a core body temperature (*T*_b_) ≤ 10 °C. One of these, hibernation, is a regular part of the annual life cycle of many species, and its fundamental features are extensively documented (e.g., Hut et al. 2002; Heldmaier et al. 2004; Williams et al. 2011; Ruf and Geiser 2015). The second circumstance is accidental deep hypothermia in neonates^1^: a phenomenon that I have argued (Hill 2017) may be as instructive as hibernation, although it has thus far been studied only indirectly. Many species of small mammals give birth to small, altricial young in seasons cold enough to subject the young to deep hypothermia if they are not kept steadily warm by their parents (Eriksson 1984; Hansson 1984; Negus and Berger 1998; Millar 2001; Stevenson et al. 2009; Duchesne et al. 2011). The implications have probably been most clearly defined in the white-footed mouse (*Peromyscus leucopus*), a New World mouse frequently abundant in woodlands of the eastern and central United States and adjacent Canada (Bedford and Hoekstra 2015). During each reproductive season in northern regions, female *P. leucopus* start to give birth to litters in the earliest days of spring (Long 1973; Millar et al. 1979; Millar and Gyug 1981), when atmospheric air temperatures below 0 °C are common, snow often falls, some soil layers remain frozen, and days of freezing weather often alternate with days of thawing weather (Long 1973). During thaws, large volumes of ice-cold melt water, flowing along paths of least resistance, may seep into the forest floor. It seems inevitable that early-born litters of *P. leucopus* are sometimes besieged by near-freezing conditions in their nests. Mothers keep litters warm when present (Hill 1972). However, two studies demonstrate that parents are away from their litters of young for many hours per day (Hill 1972; Harland and Millar 1980). During such parental absences, a neonate in its nest may experience deep hypothermia (Hill 1976). Although neonates become unresponsive to external stimulation during such hypothermia – and often stop inhaling and exhaling – their mother can restore them to their ordinary state because the only requirement for neonates to recover from deep hypothermia is rewarming (Hill et al. 2025). Moreover, *P. leucopus* nestlings that are ≤ 10 days old (ages that precede major steps in the development of homeothermy) are strikingly tolerant of deep hypothermia. Exposed to a *T*_b_ of 2–7 °C for 2–3 h, essentially all such nestlings spontaneously recover when rewarmed and go on to complete their development through to weaning (Hill 2017; Hill et al. 2025). Exposed to a *T*_b_ of 0–1 °C (without freezing), 89% survive (Hill 2017)^2^.

In both categories of mammals that undergo natural deep hypothermia, hibernators and neonates, a critical question is whether individuals’ experience of deep hypothermia alters their future ecological success. Hibernation has well-established advantages: It reduces both energy expenditure (Heldmaier and Ruf 1992; Heldmaier et al. 2004; Ruf and Geiser 2015) and mortality risk (Turbill et al. 2011). A potential disadvantage of hibernation, however, is that the prolonged exposure of the brain to near-freezing temperatures could adversely affect post-hibernation learning capacity, memory, or other cognitive functions – rendering the animals less likely to succeed ecologically in the weeks following hibernation (Millesi et al. 2001). For small mammals living in cold regions (Millar and Gyug 1981; Eriksson 1984; Hansson 1984; Negus and Berger 1998; Millar 2001; Stevenson et al. 2009; Duchesne et al. 2011), giving birth in the earliest days of spring can provide demographic advantages; a female that gives birth to an early litter is poised to maximize her number of litters over the full course of the summer breeding season, and the possibility even exists that youngsters in her initial litter will themselves be able to reproduce before summer ends (Millar et al. 1979; Gyug and Millar 1981; Millar and Gyug 1981). Birthing in the earliest days of spring, however, increases the risk of deep hypothermia in the young. As earlier noted, nestlings are highly likely to survive an episode of deep hypothermia if their mother rewarms them (Hill 2017; Hill et al. 2025). Nonetheless, in the natural world, survival might be of little consequence if the affected youngsters are deficient in learning capacity, memory, or other cognitive functions.

Since the time in the 1950 s when the first studies were carried out on cognitive effects of deep hypothermia in mammals (Andjus et al. 1955, 1956), biologists and psychologists have pursued such studies for three complementary but distinctly different reasons: ecological/evolutionary, neurological/psychological, and clinical^3^. Researchers with an ecological/evolutionary perspective have typically had a two-fold focus; first, they have focused on hypothermia that occurs during animals’ normal life histories, and second, they have focused on the potential impacts of such hypothermia on post-recovery food acquisition, predator evasion, conspecific recognition, reproductive success, and other variables of immediate ecological/evolutionary importance. Researchers with a neurological/psychological perspective have employed manipulations of brain temperature to probe brain mechanisms, such as the time course of memory formation (e.g., Ricco and Stikes 1969; Nagy et al. 1976; Nagy and Martin 1993; Palchykova et al. 2006). Researchers with a clinical perspective have sought insight into the clinical treatment of humans subjected to accidental hypothermia (e.g., Grueskin et al. 2007).

This review emphasizes the ecological/evolutionary perspective. I consider papers that have a neurological/psychological or clinical perspective, but generally incorporate here only those findings that are of ecological/evolutionary relevance. Being concerned with hypothermia that occurs during animals’ normal life histories, I do not generally include studies of adults (or relatively mature young) of non-hibernating species (e.g., laboratory rats) forced into deep hypothermia by laboratory manipulations. I also omit studies in which pharmacological agents were used to manipulate *T*_b_. Given that my concern is with deep hypothermia (*T*_b_ ≤ 10 °C), I generally do not incorporate studies in which milder hypothermia was investigated. In studies of neonatal deep hypothermia, species vary in how cold nestlings can become within the deep-hypothermia range and survive (Hill 2017). For each species my concern here is exclusively with nestlings that survive the immediate experience of deep hypothermia, regardless of which part of the deep-hypothermia range they survive. I omit studies with very small sample sizes. For example, although Pollard’s (1964) study of hedgehogs was probably the first study of the relation between hibernation and post-hibernation cognition, I do not explore the study in any detail because only three hedgehogs were studied (and for only 5 days of hibernation).

## Hibernation: experimental results

Direct brain studies of hibernating mammals have revealed changes in the brain that might, in themselves, suggest negative impacts of hibernation on cognitive function. Electroencephalographic records point to very little neural activity in the brain during hibernation (Daan et al. 1991; Arendt and Bullmann 2013). In the hippocampus, a brain region playing central roles in learning and memory formation (e.g., spatial learning), many reversible cellular effects are observed during hibernation: Key neurons are reduced in size, dendritic spines are reduced, and the types of dendritic spines are altered (Popov et al. 1992; Kirov et al. 2004; Magariños et al. 2006; Von der Ohe et al. 2006; Weltzin et al. 2006; Popov et al. 2007; Von der Ohe et al. 2007; Bullmann et al. 2016; León-Espinosa et al. 2016; Horowitz and Horwitz 2019; Hensleigh et al. 2022).

Recognizing all these potentially negative indicators, perhaps the most striking discovery of empirical cognitive research has been that – based on several studies – hibernators often emerge from hibernation with unaltered cognitive function. Millesi et al. (2001) found that individual European ground squirrels (*Spermophilus citellus*) retained their memory of other specific individuals (i.e., they exhibited social recognition) after a full winter of hibernation (early October to spontaneous termination of hibernation in the spring). Clemens et al. (2009) found that, following hibernation for the full length of their hibernation season, alpine marmots (*Marmota marmota*) retained two different behavioral maneuvers that they had learned to associate with provision of food through pre-hibernation operant conditioning. The marmots also retained a habituated, non-exploratory response to an open-field arena^4^ with which they had become highly familiar prior to hibernation. Ruczynski and Siemers (2011) trained bats (*Myotis myotis*) during pre-hibernation operant conditioning to choose a particular option in a three-choice maze to obtain food; the bats retained memory of the rewarded option after 10 weeks of hibernation. After Belding’s ground squirrels (*Urocitellus beldingi*) had emerged from hibernating for their full hibernation season, Mateo and Johnston (2000) presented them with oral-gland odors from pairs of conspecifics with which they had lived prior to hibernation. The two odor donors in each test differed in their hereditary relationship to the odor-processing animal, one donor being a littermate (close kin), the other being unrelated. The post-hibernation odor-processing ground squirrels were able to distinguish the close kin from the unrelated individuals. Hensleigh et al. (2022) studied spatial learning and memory in golden-mantled ground squirrels (*Callospermophilus lateralis*) in a circular arena (with extra-maze visual cues) equipped with multiple peripheral exit holes, only one of which permitted escape from the arena. The ground squirrels varied in performance, but the investigators emphasized that a subset of individuals, having learned to achieve rapid escape prior to hibernation, consistently maintained excellent performance during once-per-month tests during 6 months of the hibernation season (demonstrating, in the investigators’ words, that use of deep hypothermia “does not influence spatial memory in hibernating golden-mantled ground squirrels” and “there are no long-term spatial memory deficits simply as a result of torpor use in hibernation”). Bullmann et al. (2016) permitted golden (Syrian) hamsters (*Mesocricetus auratus*), prior to hibernation, to learn navigation in a custom-built maze [16 interconnected chambers (with several dead ends) through which animals traveled from a start position to a food reward] surrounded with multiple extra-maze visual cues; hibernation for 3 months did not affect the spatial memory of the hamsters.^5^

Nonetheless, hibernation sometimes degrades cognitive performance. In fact, Millesi et al. (2001) were motivated to undertake their just-mentioned formal study of cognition in European ground squirrels (*S. citellus*) by their subjective impression that the free-living ground squirrels at their outdoor field-study site underwent cognitive deterioration during the months they hibernated. Experienced squirrels at the study site accommodated to the presence of human investigators each summer. By the end of summer, when the summer ground squirrels ran away, they did not run far (< 2 m) and often begged for food. After a winter of hibernation, however, when the ground squirrels ran away, they ran ≥ 14 m.

A notable feature of Millesi et al.’s formal study (Millesi et al. 2001) was that it documented that some cognitive attributes may be degraded by hibernation while simultaneously others are not degraded, in a single species. As noted earlier, Millesi et al. (2001) found that individual European ground squirrels (*S. citellus*) retained their memory of other specific individuals after a winter of hibernation. However, the ground squirrels simultaneously underwent reductions in memory retention and cognitive performance in two learning tasks. In a spatial memory task, the animals navigated a complex tubular maze with many Y-shaped decision options, driven by a food reward. In an operant conditioning task, the animals pushed a lever to move prospective food rewards to a spot where they could be accessed. Animals were trained on the tasks prior to hibernating. Then, for the duration of the following hibernation season, whereas half the animals were permitted to hibernate, the other half were placed at an ambient temperature that prevented hibernation. The hibernating squirrels underwent clear reductions in performance in both learning tasks, compared with the non-hibernating ones, indicating that hibernation reduced memory retention.^6^

Studying Belding’s ground squirrels (*U. beldingi*), Mateo and Johnston (2000) also obtained evidence that, in a single species, some cognitive attributes may be altered by hibernation while simultaneously others are not. As described earlier, after the ground squirrels had emerged from hibernating for the full duration of their hibernation season, the investigators presented them with oral-gland odors from pairs of conspecifics. The post-hibernation odor-processing ground squirrels were able to distinguish littermates from unrelated individuals with which they had lived before. However, the post-hibernation odor-processing ground squirrels could not distinguish familiar and entirely strange individuals, i.e., ground squirrels that had been part of their social group prior to hibernation and ones they had never met before. This was true even though the squirrels could distinguish familiar and strange individuals prior to hibernation. During hibernation, they lost their memory of which conspecifics had been familiar to them.

Studying free-living Bechstein’s bats (*Myotis bechsteinii*) in the wild, Hernándes-Montero et al. (2020) found that, when the bats hibernated, they lost memory of a learned association. In the forest where the bats spent their active months, nest boxes suitable as day roosts were installed and labeled with distinctive echo-reflecting tags while, simultaneously, unsuitable nest boxes with blocked entry ways were installed and labeled with alternative tags. Bats then learned to associate the suitable nest boxes with their distinctive tags. However, after hibernating for their full hibernation season in the wild, the bats gave no evidence of remembering the association, although they soon relearned it.^7^

In tests of habitat familiarity, captive chipmunks (*Tamias striatus*) also evidenced a degree of memory loss associated with hibernation (Thompson 2011; Thompson et al. 2013). Individual chipmunks varied considerably in the amount of time they spent in hibernation, providing an opportunity to compare individuals displaying extensive hibernation with ones displaying minimal hibernation in a single species. All chipmunks were given preliminary experience with an open field prior to hibernation. When they were aroused in the middle of their hibernation season and released into that open field for 90 s, individuals that had hibernated the least showed highly evident habituation between the initial 30 s and final 30 s of their test period. In contrast, those that had hibernated the most exhibited significantly less habituation, suggesting that they were less effective in remembering the open field (or that their sensory awareness of their environment was impaired).

## Hibernation: overview

Most studies carried out on hibernators have focused on the possibility that hibernation might cause loss of memory. Prior to hibernation, studied animals have been trained to perform tasks by operant conditioning, or their ability to distinguish individuals or environmental features has been measured, or their learned ability to solve a maze problem has been quantified, or the like. Then their ability to perform the same tasks has been measured after an extended period of hibernation (either the full duration of the hibernation season or a substantial part of it). Some published papers, such as Bullmann et al. (2016), report a single experiment. Other papers report two or more experiments; Millesi et al. (2001), for example, report results of experiments on social recognition, an operant conditioning task, and a maze-learning task. A simple tally of the results of experiments produces the following result: In eight experiments, hibernators performed as well after hibernation as they performed before hibernation; that is, hibernators evidenced no loss of memory during hibernation. In five experiments, however, hibernators performed in a inferior manner after hibernation, compared with before [or, in one case (Thompson et al. 2013), animals that spent a long time in hibernation performed in an inferior manner, relative to animals that spent a short time, in a post-hibernation test of open-field habituation].

Notably, Wilkinson et al. (2017), in the first study of effects of brumation on cognitive function in a poikilothermic vertebrate, obtained results commensurate with those obtained in the majority of experiments on mammalian hibernators. After 100 days at a brumation *T*_b_ of 4 °C, the studied salamanders (*Salamandra salamandra*) exhibited no loss of memory in a spatial learning task.

A significant characteristic of the body of research presently available on cognitive effects of hibernation in mammals is its idiosyncratic quality. Speaking only a little bit loosely, every study thus far completed on mammals has employed a different method from every other study. Moreover, essentially all studies have used different species from each other, the only exception being that *S. citellus* was studied both in the early (and difficult to interpret) study by Mihailović et al. (1968; see footnote 6) and that by Millesi et al. (2001). Never has a study been designed with the deliberate goal of clarifying specific ambiguities identified in a previous study.

Although this idiosyncratic quality could be considered a weakness, it is certainly understandable. Studies of hibernation must of necessity be carried out on relatively long time scales and with great attention paid to the seasonally varying husbandry requirements of the animals. Investigators thus develop strong practical commitments to the species they study; and when an investigation is planned, the methods are often chosen to be suitable for the species, rather than vice versa. To illustrate with an example from my own research on white-footed mice (*P. leucopus*) – research focused on neonatal questions rather than hibernation (see later) – I once built a superbly crafted eight-arm radial-arm maze (Olton and Samuelson 1976) to test spatial learning in mice after they had matured from being neonates to being post-weanlings. The post-weanling *P. leucopus*, however, seemed to have no concept of what to do in a radial-arm maze; all they did was run rapidly wherever they could go. To study spatial learning in *P. leucopus*, I needed to slow the animals down and thus turned to a water maze, which yielded valuable results, as I describe later. Not that a water maze is an all-purpose solution. Bullmann et al. (2016), in their study of hibernation in golden hamsters (*M. auratus*), found that a water maze was useless because the hamsters could not swim, and nor did the hamsters function well in a radial-arm maze. Bullmann et al. (2016) therefore used a still different type of maze composed of 16 interconnected chambers, some of which acted as dead-ends as test animals attempted to move from a start position to a final position where they obtained a food reward. Hensleigh et al. (2022), in their study of hibernation in golden-mantled ground squirrels (*C. lateralis*), were particularly impressed with the proclivity of the squirrels to escape into holes when faced with predation risk. Thus Hensleigh et al. used a maze with holes, which the animals needed to enter to escape the maze. These three studies illustrate that researchers have justly prioritized compatibility between species and method, helping to explain why all the studies of hibernators have used different methods.

To integrate the results of experiments on hibernation into a fully ecological framework, an important task for future research will be to refine knowledge of how long any negative effects last. Thompson (2011) and Thompson et al. (2013) argued that chipmunks (*T. striatus*) may be able reverse negative effects of hibernation within 1–2 days after hibernation ends. Indeed, they hypothesized that the reason their studies with a radial-arm maze following hibernation were inconclusive – whereas they observed clear hibernation effects in open-field tests – was that the tests in the radial-arm maze were administered a day later than the open-field tests, providing an additional day for the chipmunks to repair negative effects of hibernation. In sharp contrast, Millesi et al. (2001) waited 4 weeks after the end of hibernation to start their post-hibernation tests on European ground squirrels (*S. citellus*), and still they observed reduced memory retention and cognitive performance in both a maze task and an operant conditioning task. At that point, Millesi et al. (2001) provided the post-hibernation ground squirrels with the opportunity to relearn the tasks, and the animals in fact fully relearned both. Relearning has usually not been included in studies of hibernators, although Hernándes-Montero et al. (2020) included relearning in their study of wild bats (*M. bechsteinii*), and they found that the post-hibernation bats – having forgotten the association between echo-reflecting tags and suitable nest boxes – promptly relearned it. From what we know (Millesi et al. 2001; Hernándes-Montero et al. 2020), negative effects of hibernation are not permanent. But to what degree do the negative effects jeopardize survival and reproduction? One requirement for answering this question will be to gain a better understanding of the full time course of the negative effects observed.

As already noted, when investigators have studied two or more cognitive functions in a single species, the results have sometimes revealed dramatic differences in the effects of hibernation on the functions studied – as when Millesi et al. (2001) found that European ground squirrels (*S. citellus*) retained their memory of other conspecific individuals following hibernation but displayed degraded memory of an operant conditioning task. As Millesi et al. (2001) explained, it is logical in such cases to postulate that the two functions are mediated by different neuronal pathways, one pathway affected in some critical way by hibernation, the other not. We can perhaps imagine a future in hibernation research in which synergistic advances are achieved by (i) using hibernation as a means of identifying distinct neuronal pathways and (ii) using direct study of neuronal pathways to better understand hibernation.

## Neonatal hypothermia: experimental results

In terms of overall perspective, most studies of neonates have differed in a major way from most studies of hibernators. As just highlighted, the central question in research on hibernators has been, “Does deep hypothermia in hibernation cause animals to have lessened memory of relationships or associations they learned prior to hibernation?” By contrast, the central question in research on neonates has been, “Does the experience of deep hypothermia during animals’ neonatal period diminish their capacity, later in life, to learn ecologically relevant relationships and associations?”

Hill and colleagues have studied this question in white-footed mice, *P. leucopus*, in three research reports, in all of which the test animals were exposed to four 2.5- to 3-h-long episodes of deep hypothermia (*T*_b_ ≅ 2–3 °C) when 3–10 days old. The cognitive abilities of the animals were then assessed, relative to control littermates, after they had developed sufficiently to be weaned and live independently of their parents.

Hill and Eshuis (1988) studied the performance of mice that had reached 2–6 months of age in two cognitive tasks: spatial learning in a Morris water maze (Morris 1981; Sutherland and Dyck 1984; Young et al. 2006) and taste-aversion learning (Robbins 1978). The water maze, surrounded with extra-maze visual cues, provided a test of the ability of the mice to learn place navigation as they discovered that a slightly underwater escape platform was present and how to find it. Each hypothermia-treated animal and a littermate control animal were observed in the maze every day for 8 consecutive days. Over this period, as learning occurred, there were no statistically significant differences between the hypothermia-treated and control mice in latency (time required to find the escape platform), failure (proportion of animals that did not find the platform), or ability to re-find the platform after it had been moved to a new location. In the taste-aversion test, animals’ first experience of sucrose in their drinking water was paired with mild lithium poisoning (causing the animals to develop an aversion to the taste of sucrose), but thereafter they were given safe sucrose-flavored water without lithium (Robbins 1978). The hypothermia-treated and control mice, when studied every other day for 18 days following acquisition of taste aversion, did not differ significantly in either the initial magnitude of their aversion to sucrose or the extinction of the aversion.

Hill et al. (2008) carried out a second study of place-navigation learning using the Morris water maze. This study was designed to be methodologically superior to the earlier study in three ways. (i) The sample size was larger: 68 pairs of mice versus 47 pairs in the earlier study (each pair composed of a hypothermia-treated individual and littermate control). (ii) Testing in the maze was started sooner after weaning: 1–2 weeks after weaning, rather than ca. 1–5 months after. (iii) Trials were video recorded, permitting calculation of additional performance metrics, such as the total distance animals swam as they searched for and found the escape platform. Studied as weanlings, the hypothermia-treated mice in these trials exhibited no statistically significant differences from control littermates in latency, failure rate, distance traveled, or speed as they progressively learned the location of the escape platform in 7 successive days of experience in the maze. With video recording, Hill et al. (2008) were also able to carry out revealing experiments, termed *probe trials* in the psychology literature, in which, after mice had learned the position of the escape platform, the platform was removed, and the mice were tested with no platform present. In these trials, mice tended to swim repeatedly over the position where the platform had been, and there was no significant difference between the hypothermia-treated and control siblings in number of passes over the platform location during a standardized time interval. Following the probe trials, the mice were again tested with the platform present on four occasions over the ensuing 4 weeks. Again, the hypothermia-treated mice exhibited no statistically significant differences from control littermates.

Ulanovsky (2025) has recently argued for the unique virtues of naturalistic experiments. Hill (2017) carried out such an experiment, in which pairs of *P. leucopus* (hypothermia-treated and littermate control) were subjected to predation by screech owls (*Megascops asio*) in a room-size arena (floor area: 9.9 m^2^) designed to mimic woodland habitat. Each pair of mice (at 43–83 days of age) lived in the arena for 1–3 days before an owl was permitted to enter the arena in the night. After arrival of the owl, the arena was observed continually until the owl captured one of the two mice. Based on results for six owls that collectively preyed on 74 pairs of mice, there was no evidence that the hypothermia-treated mice were inferior to control siblings in their ability to evade capture.

The motivation for carrying out the studies on *P. leucopus* was entirely ecological/evolutionary, focusing on the question of whether mice living in the wild might go through their lives with cognitive deficits if they experienced deep hypothermia as neonates. There has been a second, more pragmatic motivation for conducting research on the potential cognitive effects of neonatal deep hypothermia, namely the drive to assess whether cryoanesthesia has unintended cognitive effects. Cryoanesthesia – the use of deep hypothermia to induce general anesthesia (Phifer and Terry 1986) – has been used for decades as an often-preferred method for inducing anesthesia in neonates for research surgeries (Phifer and Terry 1986; Kolb and Cioe 2001; Richter et al. 2014; Janus and Golde 2014). In 1998, however, Nuñez et al. (1998) announced that neonatal cryoanesthesia could alter the morphology of the visual cortex in laboratory rats (*Rattus norvegicus*) – a finding that opened up the possibility that effects of cryoanesthesia could sometimes confound results in a wide range of experiments on rodent behavior in which the animals were subjected to surgery (e.g., gonadectomy) as neonates.

A problem for reviewing the cryoanesthesia literature in the present context is that researchers – while employing obviously useful techniques for inducing deep hypothermia (e.g., placing neonates in contact with ice or immersing them in a slush of water and ice) – have often not measured *T*_b_, meaning that the hypothermia might not always have qualified as *deep* hypothermia.^8^ Another preliminary point to note is that episodes of hypothermia during cryoanesthesia have typically been brief, durations of 5–15 min being the norm.

Nuñez et al. (2000) followed up on their earlier report (Nuñez et al. 1998) with a study of laboratory rats in which they included cognitive tests in a Morris water maze. Neonatal rats, on their day of birth, were subjected to a single 60-min period of hypothermia (unusually long for cryoanesthesia; *T*_b_ not directly measured). Then, when the rats had reached about 140 days of age, they were tested in the water maze. At the conclusion of the sequence of learning trials in the maze, the hypothermia-treated and control rats were identical in their knowledge of the location of the escape platform, as judged by probe trials. However, based on measures of latency and distance traveled during the learning trials, the hypothermia-treated rats had been slower to learn the location of the platform. Kolb and Cioe (2001) promptly repeated the study by Nuñez et al. (2000) with a focus on 10-day-olds as well as 1-day-olds. Neonatal laboratory rats of both ages were subjected to a 9- to 14-min period of cryoanesthesia (*T*_b_ not directly measured). Then, when about 4 months old, they were tested in a Morris water maze. For rats subjected to hypothermia at 1 day of age, Kolb and Cioe (2001) obtained results that resembled those of Nuñez et al. (2000), confirming a negative effect of neonatal hypothermia on the rate of learning^9^. However, Kolb and Cioe (2001) found that rats subjected to hypothermia when 10 days old exhibited no differences from controls in the maze tests at 4 months of age, suggesting that in laboratory rats neonatal cryoanesthesia causes adult effects only when the neonates are very young.

Janus and Golde (2014) investigated laboratory mice (*Mus musculus*) that, on the day they were born, were subjected to cryoanesthesia for 6–12 min (skin surface *T*_b_ fell to 5–8 °C; deep *T*_b_ not measured). After the mice reached 4–5 months of age, they were subjected to two cognitive tests. In a Morris water maze, the cryoanesthesia-treated mice did not differ significantly from controls in their acquisition of successful place navigation; nor did they differ in their performance during probe trials. The mice at 4–5 months of age were also subjected to an operant conditioning task. They were presented twice with mild electrical shocks simultaneously with an auditory tone. Subsequently, when they were presented with just the tone, the cryoanesthesia-treated and control mice exhibited no significant difference in their learned display of aversion (locomotory stand-still).

Richter et al. (2014) studied laboratory mice subjected to cryoanesthesia for 5 min when 3 days old (*T*_b_ not directly measured). When the mice reached 84–130 days of age, they and controls were compared in eight different standardized tests designed to measure a diversity of behaviors such as exploratory behavior, nesting behavior, response to conflicting motivations, response to an unsolvable problem, and response to pain. The cryoanesthesia-treated mice exhibited no significant differences from control mice in any of these tests (or in serum corticosterone concentration), except for a small increase in a measure of anxiety in one test.

## Neonatal hypothermia: overview

An important observation is that – in the species of rodents studied – after neonates have been exposed to deep hypothermia and returned to their parents, the neonates typically continue their nestling development and are successfully weaned. I have quantified this process in *P. leucopus*. Among 253 nestlings treated with four 2.5-h episodes of deep hypothermia as neonates, 94% survived to the age of weaning in the care of their parents, a survivorship not statistically different from that of control siblings not exposed to neonatal hypothermia (Hill 2017). Moreover, the growth of deep-hypothermia-treated *P. leucopus* neonates during their nestling life is approximately normal (Hill et al. 2008): When measured in early maturity, nestlings subjected four times to deep hypothermia at 4–10 days of age were found to be identical in body length to control littermates (although 7% lower in body weight).

Nestlings have cognitive processes – which I here term “within-nest” cognitive processes – that are important during their nestling lives, as they interact with each other and their parents (e.g., nursing) during development (e.g., Wiedenmayer et al. 2000; Armstrong et al. 2006; Meyer and Alberts 2016). In principle, neonatal deep hypothermia could exert very *immediate* cognitive effects by impacting critical within-nest cognitive processes. However, no direct research on this possibility has ever been published, in any species. The high survivorship of hypothermia-treated youngsters during their nestling life – and their relatively ordinary growth and morphological development – suggest that their within-nest cognitive processes remained intact despite the hypothermia they experienced in their first days of life. Direct studies of deep-hypothermia effects on within-nest cognitive processes are needed, however^10^.

In contrast, as seen in the preceding section, several studies have addressed the potential *long-term* cognitive effects of neonatal deep hypothermia, i.e., cognitive effects evident after the nestlings have matured to become post-weanlings or adults. Collectively, these studies indicate that neonatal deep hypothermia typically has little or no effect on post-weaning or adult cognition, although the number of species studied (three) has been limited and each study has addressed just a limited set of cognitive processes.

We sometimes say that nestling rodents “tolerate” neonatal deep hypothermia, or that they endogenously “recover” from it. These words can have two different meanings. In a limited and short-term sense, the words can simply mean that the animals are not killed by neonatal deep hypothermia. From an ecological/evolutionary perspective, however, the substantive question is whether the nestlings can mature into ordinary, cognitively normal adults despite having experienced deep hypothermia as neonates. Available data indicate that in general they can do so. That is, nestling rodents in general “tolerate” neonatal deep hypothermia in the substantive, ecologically relevant sense. The one known exception is the slowing of learning that Nuñez et al. (2000) and Kolb and Cioe (2001) observed in 4- to 5-month-old laboratory rats that had been exposed to deep hypothermia as 1-day-olds, an effect described as “small” by Kolb and Cioe (2001)^11^.

## Concluding perspectives

Despite doubts in the past (Hill 2000), the evidence today says that both hibernating mammals (Heldmaier et al. 2004; Ruf and Geiser 2015) and neonates in deep hypothermia (Fitzgerald 1955; Hill et al. 2025) consume O_2_ steadily and function aerobically. In fact, the rate of O_2_ consumption by neonatal *P. leucopus* in deep hypothermia is the same as the rate of O_2_ consumption by hibernators of similar body size in hibernation (Hill et al. 2025). The fact that both groups of animals are aerobic undoubtedly helps explain why neither group suffers catastrophic cognitive failure following deep hypothermia, and it probably means that in cases where cognitive deficits are found, future research will reveal commonalities in the causation of the deficits in the two groups.

Based on the information available today, and taking a coarse statistical approach, the odds are better than 50:50 that hibernators will emerge from each year’s hibernation season without measurable cognitive deficits. Neonates that experience deep hypothermia will generally emerge without measurable cognitive deficits, although even a brief experience of deep hypothermia at a critical stage of development might leave a small cognitive deficit in its wake.

From an ecological/evolutionary point of view, one of the great remaining uncertainties is the question of how rapidly and completely hibernators and matured neonates that have incurred cognitive deficits can make up for the deficits through repair, compensation, or learning (e.g., relearning of past associations). Even if deficits occur, their ecological/evolutionary impact will be minimal when the deficits can be negated rapidly.

Interestingly, Harris et al. (2004) have hypothesized that hibernation in adult mammals might be a trait that is evolutionally descended from the proclivity of neonates to undergo large variations in *T*_b_. This hypothesis, if supported, might help explain why hibernators experiencing deep hypothermia seem to resemble neonates in being relatively resistant to suffering cognitive damage.

Existing research on the effects of deep hypothermia on cognitive abilities has probed the performance of hypothermia-experienced animals in a wide range of cognitive tasks: spatial learning (using at least four types of mazes), social recognition, responses to aversive stimuli, predator avoidance, and operant conditioning processes. In essence, existing research has cast a wide net, which has obvious advantages from a natural-history point of view and also from the perspective of leaving no stone unturned. However, for future progress in understanding the fundamentals of hibernation and hypothermia effects on cognition, researchers may collectively need to choose and pursue a limited set of problems and models to gain the benefits of focus.

## Footnotes

1 I define neonates to be young nestlings, or more specifically, nestlings that are half the age of weaning or younger. The age of weaning is considered to be about 21 days in many rodent species. Neonates in these species are nestlings that are 10–11 days old or younger.

2 The neonates of a diversity of species, in addition to *P. leucopus*, have been documented to tolerate at least some *T*_b_s in the range *T*_b_ ≤ 10 °C for at least 0.5 h, including *Mesocricetus auratus* (golden hamster; Adolph 1951; Corcoran et al. 2012), *Microtus ochrogaster* (prairie vole; Hill 2017), *Mus musculus* (house mouse, laboratory mouse; Fitzgerald 1955; Hill 2017), *Oryctolagus cuniculus* (domestic rabbit; Adolph 1951), *Peromyscus melanophrys* (plateau mouse; Hill 2017), *Rattus norvegicus* (brown rat, laboratory rat; Adolph 1951; Tattersall and Milsom 2003; Hill 2017), *Sigmodon hispidus* (hispid cotton rat; Scheck 1982), and *Felus catus* (domestic cat; Adolph 1951).

3 Mrosovsky's (1967) excellent, but now dated, review – the only prior review – was almost entirely concerned with the neurological/psychological and clinical perspectives, in part because almost no information on cognitive function in hibernators existed at the time.

4 In an open-field test, an animal is placed in an open arena with visual markings but no barriers or rewards, and the animal’s exploratory movements are recorded. Animals typically move around in the arena to a great extent at first but gradually move around less and less as they are provided with repeated experiences in the open field – a pattern interpreted to mean that they progressively learn about the nature of the field and become less motivated to explore it (i.e., “habituated”).

5 Pollard (1964), although reporting no negative cognitive effects, is omitted as earlier noted, because of small sample size (3 animals) and brief hibernation duration (5 days). McNamara and Riedesel (1973), although also reporting no negative effects, is omitted because – in view of criticism (Alloway 1973; Millesi et al. 2001) – the meaning of the reported results is unclear.

6 European ground squirrels (*S. citellus*) were also studied by Mihailović et al. (1968) in one of the earliest papers on hibernation and cognition. Interpretation of the results of that study is hindered by the fact that the authors reported only incomplete measures of individual variation, as Millesi et al. (2001) also noted. The one conclusion in Mihailović et al. (1968) that seemed to be well supported by the data reported was that animals recently emerged from hibernation learned a maze task more rapidly than ones without an immediately preceding hibernation experience.

7 Henke-von der Malsburg et al. (2023) also attempted to study memory in the wild, focusing on grey mouse lemurs (*Microcebus murinus*) in Madagascar. Their informative study is not discussed here because they concluded that observed memory loss was likely not attributable to hibernation, and body temperatures of hibernating animals may not have qualified as being deep hypothermia.

8 In their paper defining cryoanesthesia, Phifer and Terry (1986) demonstrated by direct measurement that the rectal *T*_b_ of 6-day-old laboratory rats fell below10°C in about 4 min when the rats were immersed in a slush of water and ice. This finding perhaps suggested to subsequent researchers that direct *T*_b_ measurement is unnecessary even when exposure to cryoanesthesia conditions is brief.

9 In deep-hypothermia survivorship tests by Hill (2017), neonatal laboratory rats required *T*_b_s several degrees Celsius higher than neonatal *P. leucopus* to survive. This result at least superficially parallels the fact that in the tests in a Morris water maze discussed here, laboratory rats evidenced a negative cognitive effect of neonatal deep hypothermia, whereas when *P. leucopus* were studied in a Morris water maze (see earlier), they exhibited no effects of neonatal deep hypothermia even though the *P. leucopus* neonates were exposed to a far longer, deeper experience of hypothermia than the rat neonates.

10 There is, however, at least one published study of the effects of brief (1 min), mild (*T*_b_ ≅ 17 °C) hypothermia on a cognitive process in nestling laboratory mice (Nagy et al. 1976).

11 This is a narrowly defined vulnerability. Laboratory mice exposed to hypothermia as 1-day-olds were not affected (Janus and Golde 2014), and laboratory rats exposed as 10-day-olds were not affected (Kolb and Cioe 2001).

## References

[CR1] Adolph EF (1951) Responses to hypothermia in several species of infant mammals. Am J Physiol 166:75–91. 10.1152/ajplegacy.1951.166.1.75 14857150 10.1152/ajplegacy.1951.166.1.75

[CR2] Alloway TM (1973) Hibernation: effects on memory or performance? Science 181:86–87. 10.1126/science.181.4094.86 4714295 10.1126/science.181.4094.86

[CR3] Andjus RK, Knöpfelmacher F, Russell RW, Smith AU (1955) Effects of hypothermia on behaviour. Nature 176:1015–1016. 10.1038/1761015a0 13272741 10.1038/1761015a0

[CR4] Andjus RK, Knöpfelmacher F, Russell RW, Smith AU (1956) Some effects of severe hypothermia on learning and retention. Q J Exp Psychol 8:15–23. 10.1080/17470215608416797

[CR5] Arendt T, Bullmann T (2013) Neuronal plasticity in hibernation and the proposed role of the microtubule-associated protein Tau as a master switch regulating synaptic gain in neuronal networks. Am J Physiol 305:R478–R489. 10.1152/ajpregu.00117.2013 10.1152/ajpregu.00117.2013 23824962

[CR6] Armstrong CM, DeVito LM, Cleland TA (2006) One-trial associative odor learning in neonatal mice. Chem Senses 31:343–349. 10.1093/chemse/bjj038 16495436 10.1093/chemse/bjj038

[CR7] Bedford NL, Hoekstra HE (2015) The natural history of model organisms: *Peromyscus* mice as a model for studying natural variation. elife 4:e06813. 10.7554/eLife.06813 26083802 10.7554/eLife.06813 PMC4470249

[CR8] Bullmann T, Seeger G, Stieler J, Hanics J, Reimann K, Kretzschmann TP, Hilbrich I, Holzer M, Alpár A, Arendt T (2016) Tau phosphorylation-associated spine regression does not impair hippocampal-dependent memory in hibernating golden hamsters. Hippocampus 26:301–318. 10.1002/hipo.22522 26332578 10.1002/hipo.22522

[CR9] Clemens LE, Heldmaier G, Exner C (2009) Keep cool: memory is retained during hibernation in alpine marmots. Physiol Behav 98:78–84. 10.1016/j.physbeh.2009.04.013 19393672 10.1016/j.physbeh.2009.04.013

[CR10] Corcoran AE, Andrade DV, Marshall LH, Milsom WK (2012) Developmental changes in cold tolerance and ability to autoresuscitate from hypothermic respiratory arrest are not linked in rats and hamsters. Respir Physiol Neurobiol 181:249–258. 10.1016/j.resp.2012.03.006 22450012 10.1016/j.resp.2012.03.006

[CR11] Daan S, Barnes BM, Strijkstra AM (1991) Warming up for sleep? – ground squirrels sleep during arousals from hibernation. Neurosci Lett 128:265–268. 10.1016/0304-3940(91)90276-Y 1945046 10.1016/0304-3940(91)90276-y

[CR12] Duchesne D, Gauthier G, Berteaux D (2011) Habitat selection, reproduction and predation of wintering lemmings in the Arctic. Oecologia 167:967–980. 10.1007/s00442-011-2045-6 21701915 10.1007/s00442-011-2045-6

[CR13] Eriksson M (1984) Winter breeding in three rodent species, the bank vole *Clethrionomys glareolus*, the yellow-necked mouse *Apodemus flavicollis* and the wood mouse *Apodemus sylvaticus* in southern Sweden. Holarctic Ecol 7:428–429. 10.1111/j.1600-0587.1984.tb01144.x

[CR14] Fitzgerald LR (1955) Oxygen consumption of newborn mice at low temperatures. Am J Physiol 182:105–110. 10.1152/ajplegacy.1955.182.1.105 13248952 10.1152/ajplegacy.1955.182.1.105

[CR15] Grueskin J, Tanen DA, Harvey P, Dos Santos F, Richardson WH, Riffenburgh RH (2007) A pilot study of mechanical stimulation and cardiac dysrhythmias in a porcine model of induced hypothermia. Wild Environ Med 18:133–137. 10.1580/06-WEME-BR-034R1.1 10.1580/06-WEME-BR-034R1.1 17590058

[CR16] Gyug LW, Millar JS (1981) Growth of seasonal generations in three natural populations of *Peromyscus*. Can J Zool 59:510–514. 10.1139/z81-074

[CR17] Hansson L (1984) Winter reproduction of small mammals in relation to food conditions and population dynamics. Carnegie Mus Nat Hist Spec Publ 10:225–234

[CR18] Harland RM, Millar JS (1980) Activity of breeding *Peromyscus leucopus*. Can J Zool 58:313–316. 10.1139/z80-039

[CR19] Harris MB, Olson LE, Milsom WK (2004) The origin of mammalian heterothermy: a case for perpetual youth? In: Barnes BM, Carey HV (eds) Life in the cold: evolution, mechanisms, adaptation, and application. Biol papers, vol 27. Univ Alaska, Fairbanks, pp 41–50

[CR20] Heldmaier G, Ruf T (1992) Body temperature and metabolic rate during natural hypothermia in endotherms. J Comp Physiol B 162:696–706. 10.1007/BF00301619 1494028 10.1007/BF00301619

[CR21] Heldmaier G, Ortmann S, Elvert R (2004) Natural hypometabolism during hibernation and daily torpor in mammals. Respir Physiol Neurobiol 141:317–329. 10.1016/j.resp.2004.03.014 15288602 10.1016/j.resp.2004.03.014

[CR22] Henke-von der Malsburg J, Fichtel C, Kappeler PM (2023) Retaining memory after hibernation: performance varies independently of activity levels in wild grey mouse lemurs. Ethology 129:12–23. 10.1111/eth.13337

[CR23] Hensleigh E, Murtishaw AS, Treat MD, Heaney CF, Bolton MM, Sabbagh JJ, Calvin KN, Kinney JW, van Breukelen F (2022) Torpor does not influence spatial memory in hibernating golden-mantled ground squirrels (*Spermophilus* [*Callospermophilus*] *lateralis*). Physiol Biochem Zool 95:390–399. 10.1086/721185 35930827 10.1086/721185

[CR24] Hernández-Montero JR, Schöner CR, Kerth G (2020) No evidence for memory retention of a learned association between a cue and roost quality after hibernation in free-ranging bats. Ethology 126:761–771. 10.1111/eth.13029

[CR25] Hill RW (1972) The amount of maternal care in *Peromyscus leucopus* and its thermal significance for the young. J Mammal 53:774–790. 10.2307/1379213 4654241

[CR26] Hill RW (1976) The ontogeny of homeothermy in neonatal *Peromyscus leucopus*. Physiol Zool 49:392–306. 10.1086/physzool.49.3.30155689

[CR27] Hill RW (2000) Anoxia tolerance to oxygen necessity: paradigm shift in the physiology of survival of apneic deep hypothermia in neonatal rodents. In: Heldmaier G, Klingenspor M (eds) Life in the cold. Springer-Verlag, Berlin, pp 199–205. 10.1007/978-3-662-04162-8_21

[CR28] Hill RW (2017) Benign neonatal deep hypothermia in rodents and its relations to hibernation. J Comp Physiol B 187:705–713. 10.1007/s00360-017-1070-0 28349198 10.1007/s00360-017-1070-0

[CR29] Hill RW, Eshuis RK (1988) Learning in mature mice (*Peromyscus leucopus*) subjected to deep hypothermia as neonates. J Comp Psychol 102:44–48. 3365944 10.1037/0735-7036.102.1.44

[CR30] Hill RW, Gunn JP, Champagne AM, Cook M (2008) Capacity for spatial learning in weanling white-footed mice (*Peromyscus leucopus*) is unaffected by neonatal near-freezing hypothermia. In: Lovegrove BG, McKechnie AE (eds) Hypometabolism in animals: hibernation, torpor and cryobiology. University of KwaZulu-Natal, Pietermaritzburg, South Africa, pp 231–240

[CR31] Hill RW, Manteuffel JJ, White BA (2025) Apneic uptake of atmospheric O_2_ by deeply hypothermic nestlings of the white-footed mouse (*Peromyscus leucopus*): circulation and lungs. J Comp Physiol B 195:123–139. 10.1007/s00360-024-01585-x 39375204 10.1007/s00360-024-01585-x PMC11839856

[CR32] Horowitz JM, Horwitz BA (2019) Extreme neuroplasticity of hippocampal CA1 pyramidal neurons in hibernating mammalian species. Front Neuroanat 13:9. 10.3389/fnana.2019.00009 30814935 10.3389/fnana.2019.00009 PMC6381046

[CR33] Hut RA, Barnes BM, Daan S (2002) Body temperature patterns before, during, and after semi-natural hibernation in the European ground squirrel. J Comp Physiol B 172:47–58. 10.1007/s003600100226 11824403 10.1007/s003600100226

[CR34] Janus C, Golde T (2014) The effect of brief neonatal cryoanesthesia on physical development and adult cognitive function in mice. Behav Brain Res 259:253–260. 10.1016/j.bbr.2013.11.010 24239696 10.1016/j.bbr.2013.11.010 PMC3883048

[CR35] Kirov SA, Petrak LJ, Fiala JC, Harris KM (2004) Dendritic spines disappear with chilling but proliferate excessively upon rewarming of mature hippocampus. Neuroscience 127:69–80. 10.1016/j.neuroscience.2004.04.053 15219670 10.1016/j.neuroscience.2004.04.053

[CR36] Kolb B, Cioe J (2001) Cryoanesthesia on postnatal day 1, but not day 10, affects adult behavior and cortical morphology in rats. Dev Brain Res 130:9–14. 10.1016/S0165-3806(01)00182-1 11557089 10.1016/s0165-3806(01)00182-1

[CR37] León-Espinosa G, García E, Gómez-Pinedo U, Hernández F, Defelipe J, Ávila J (2016) Decreased adult neurogenesis in hibernating Syrian hamster. Neuroscience 333:181–192. 10.1016/j.neuroscience.2016.07.016 27436535 10.1016/j.neuroscience.2016.07.016

[CR38] Long CA (1973) Reproduction in the white-footed mouse at the northern limits of its geographical range. Southwest Nat 18:11–20. 10.2307/3669906

[CR39] Magariños AM, McEwen BS, Saboureau M, Pevet P (2006) Rapid and reversible changes in intrahippocampal connectivity during the course of hibernation in European hamsters. Proc Natl Acad Sci USA 103:18775–18780. 10.1073/pnas.0608785103 17121986 10.1073/pnas.0608785103 PMC1693738

[CR40] Mateo JM, Johnston RE (2000) Retention of social recognition after hibernation in Belding’s ground squirrels. Anim Behav 59:491–499. 10.1006/anbe.1999.1363 10715170 10.1006/anbe.1999.1363

[CR41] McNamara MC, Riedesel ML (1973) Memory and hibernation in *Citellus lateralis*. Science 179:92–94. 10.1126/science.179.4068.92 4682136 10.1126/science.179.4068.92

[CR42] Meyer PM, Alberts JR (2016) Non-nutritive, thermotactile cues induce odor preference in infant mice (*Mus musculus*). J Comp Psychol 130:369–379. 10.1037/com0000044 27599356 10.1037/com0000044 PMC5108687

[CR43] Mihailović LJ, Petrović-Minić B, Protić S, Divac I (1968) Effects of hibernation on learning and retention. Nature 218:191–192. 10.1038/218191a0 5645294 10.1038/218191a0

[CR44] Millar JS (2001) On reproduction in lemmings. Ecoscience 8:145–150. 10.1080/11956860.2001.11682639

[CR45] Millar JS, Gyug LW (1981) Initiation of breeding by northern *Peromyscus* in relation to temperature. Can J Zool 59:1094–1098. 10.1139/z81-151

[CR46] Millar JS, Wille FB, Iverson SL (1979) Breeding by *Peromyscus* in seasonal environments. Can J Zool 57:719–727. 10.1139/z79-090

[CR47] Millesi E, Prossinger H, Dittami JP, Fieder M (2001) Hibernation effects on memory in European ground squirrels (*Spermophilus citellus*). J Biol Rhyth 16:264–271. 10.1177/074873040101600309 10.1177/074873040101600309 11407786

[CR48] Morris RGM (1981) Spatial localization does not require the presence of local cues. Learn Motiv 12:239–260. 10.1016/0023-9690(81)90020-5

[CR49] Mrosovsky N (1967) Lowered body temperature, learning and behaviour. In: Fisher KC (ed) Mammalian hibernation III. American Elsevier, New York, pp 152–173

[CR50] Nagy ZM, Martin DJ (1993) Hypothermia-induced retrograde amnesia in young and adult Swiss mice. Bull Psychon Soc 31:225–228. 10.3758/BF03337330

[CR51] Nagy ZM, Anderson JA, Mazzaferri TA (1976) Hypothermia causes adult-like retention deficits of prior learning in infant mice. Dev Psychobiol 9:447–458. 10.1002/dev.420090507 986975 10.1002/dev.420090507

[CR52] Negus NC, Berger PJ (1998) Reproductive strategies of *Dicrostonyx groenlandicus* and *Lemmus sibiricus* in high-arctic tundra. Can J Zool 76:390–399. 10.1139/z97-226

[CR53] Nuñez JL, Kim BY, Juraska JM (1998) Neonatal cryoanesthesia affects the morphology of the visual cortex in the adult rat. Dev Brain Res 111:89–98. 10.1016/S0165-3806(98)00125-4 9804905 10.1016/s0165-3806(98)00125-4

[CR54] Nuñez JL, Koss WA, Juraska JM (2000) Hippocampal anatomy and water maze performance are affected by neonatal cryoanesthesia in rats of both sexes. Horm Behav 37:169–178. 10.1006/hbeh.2000.1572 10868480 10.1006/hbeh.2000.1572

[CR55] Olton DS, Samuelson RJ (1976) Remembrance of places passed: spatial memory in rats. J Exp Psychol Anim Behav Process 2:97–116. 10.1037/0097-7403.2.2.97

[CR56] Palchykova S, Crestani F, Meerlo P, Tobler I (2006) Sleep deprivation and daily torpor impair object recognition in Djungarian hamsters. Physiol Behav 87:144–153. 10.1016/j.physbeh.2005.09.005 16253296 10.1016/j.physbeh.2005.09.005

[CR57] Phifer CB, Terry LM (1986) Use of hypothermia for general anesthesia in preweanling rodents. Physiol Behav 38:887–890. 10.1016/0031-9384(86)90058-2 3823208 10.1016/0031-9384(86)90058-2

[CR58] Pollard JS (1964) Hedgehogs and the retroaction theory of forgetting. Psychol Rep 15:128–130. 10.2466/pr0.1964.15.1.128

[CR59] Popov VI, Bocharova LS, Bragin AG (1992) Repeated changes of dendritic morphology in the hippocampus of ground squirrels in the course of hibernation. Neuroscience 48:45–51. 10.1016/0306-4522(92)90336-Z 1584424 10.1016/0306-4522(92)90336-z

[CR60] Popov VI, Medvedev NI, Patrushev IV, Ignat’ev DA, Morenkov ED, Stewart MG (2007) Reversible reduction in dendritic spines in CA1 of rat and ground squirrel subjected to hypothermia-normothermia *in vivo*: a three-dimensional electron microscope study. Neuroscience 149:549–560. 10.1016/j.neuroscience.2007.07.059 17919827 10.1016/j.neuroscience.2007.07.059

[CR61] Riccio DC, Stikes ER (1969) Persistent but modifiable retrograde amnesia produced by hypothermia. Physiol Behav 4:649–652. 10.1016/0031-9384(69)90170-X

[CR62] Richter SH, Wollmann E, Schmidt M, Zillmann U, Hellweg R, Sprengel R, Gass P (2014) The effects of cryoanesthesia-induced hypothermia on adult emotional behaviour and stress markers in C57BL/6 mice. Behav Brain Res 270:300–306. 10.1016/j.bbr.2014.05.002 24814613 10.1016/j.bbr.2014.05.002

[CR63] Robbins RJ (1978) Poison-based taste aversion learning in deer mice (*Peromyscus maniculatus bairdi*). J Comp Physiol Psychol 92:642–650. 10.1037/h0077493 690287 10.1037/h0077493

[CR64] Ruczynski I, Siemers BM (2011) Hibernation does not affect memory retention in bats. Biol Lett 7:153–155. 10.1098/rsbl.2010.0585 20702450 10.1098/rsbl.2010.0585 PMC3030893

[CR65] Ruf T, Geiser F (2015) Daily torpor and hibernation in birds and mammals. Biol Rev 90:891–926. 10.1111/brv.12137 25123049 10.1111/brv.12137 PMC4351926

[CR66] Scheck SH (1982) Development of thermoregulatory abilities in the neonatal hispid cotton rat, *Sigmodon hispidus texianus*, from northern Kansas and south-central Texas. Physiol Zool 55:91–104. 10.1086/physzool.55.1.30158446

[CR67] Stevenson KT, van Tets IG, Nay LAI (2009) The seasonality of reproduction in photoperiod responsive and nonresponsive northern red-backed voles (*Myodes rutilus*) in Alaska. Can J Zool 87:152–164. 10.1139/Z08-147

[CR68] Sutherland RJ, Dyck RH (1984) Place navigation by rats in a swimming pool. Can J Psychol 38:322–347. 10.1037/h0080832

[CR69] Tattersall GJ, Milsom WK (2003) Hypothermia-induced respiratory arrest and recovery in neonatal rats. Resp Physiol Neurobi 137:29–40. 10.1016/S1569-9048(03)00112-5 10.1016/s1569-9048(03)00112-5 12871675

[CR70] Thompson AB (2011) Correlates and consequences of heterothermy in mammals. Thesis, McGill University

[CR71] Thompson AB, Montiglio P-O, Humphries MM (2013) Behavioural impacts of torpor expression: a transient effect in captive eastern chipmunks (*Tamias striatus*). Physiol Behav 110:115–121. 10.1037/h008083216/j.physbeh.2013.01.005 23313403 10.1016/j.physbeh.2013.01.005

[CR72] Turbill C, Bieber C, Ruf T (2011) Hibernation is associated with increased survival and the evolution of slow life histories among mammals. Proc R Soc B 278:3355–3363. 10.1098/rspb.2011.0190 21450735 10.1098/rspb.2011.0190 PMC3177628

[CR73] Ulanovsky N (2025) Natural neuroscience: toward a systems neuroscience of natural behaviors. MIT Press, Cambridge, Massachusetts. 10.7551/mitpress/10813.001.0001

[CR74] Von der Ohe CG, Darian-Smith C, Garner CC, Heller HC (2006) Ubiquitous and temperature-dependent neural plasticity in hibernators. J Neurosci 26:10590–10598. 10.1523/JNEUROSCI.2874-06.2006 17035545 10.1523/JNEUROSCI.2874-06.2006 PMC6674705

[CR75] Von der Ohe CG, Garner CC, Darian-Smith C, Heller HC (2007) Synaptic protein dynamics in hibernation. J Neurosci 27:84–92. 10.1523/JNEUROSCI.4385-06.2007 17202475 10.1523/JNEUROSCI.4385-06.2007 PMC6672296

[CR76] Weltzin MM, Zhao HW, Drew KL, Bucci DJ (2006) Arousal from hibernation alters contextual learning and memory. Behav Brain Res 167:128–133. 10.1016/j.bbr.2005.08.021 16219369 10.1016/j.bbr.2005.08.021 PMC12186774

[CR77] Wiedenmayer CP, Myers MM, Mayford M, Barr GA (2000) Olfactory based spatial learning in neonatal mice and its dependence on CaMKII. Neuroreport 11:1051–1055. 10.1097/00001756-200004070-00030 10790881 10.1097/00001756-200004070-00030

[CR78] Wilkinson A, Hloch A, Mueller-Paul J, Huber L (2017) The effect of brumation on memory retention. Sci Rep 7:40079. 10.1038/srep40079 28074838 10.1038/srep40079 PMC5225446

[CR79] Williams CT, Sheriff MJ, Schmutz JA, Kohl F, Tøien Ø, Buck CL, Barnes BM (2011) Data logging of body temperatures provides precise information on phenology of reproductive events in a free-living arctic hibernator. J Comp Physiol B 181:1101–1109. 10.1007/s00360-011-0593-z 21691770 10.1007/s00360-011-0593-z

[CR80] Young GS, Choleris E, Kirkland JB (2006) Use of salient and non-salient visuospatial cues by rats in the Morris water maze. Physiol Behav 87:794–799. 10.1016/j.physbeh.2006.01.022 16516936 10.1016/j.physbeh.2006.01.022

